# Correlation of mRNA Expression and Signal Variability in Chronic Intracortical Electrodes

**DOI:** 10.3389/fbioe.2018.00026

**Published:** 2018-03-27

**Authors:** Jessica D. Falcone, Sheridan L. Carroll, Tarun Saxena, Dev Mandavia, Alexus Clark, Varun Yarabarla, Ravi V. Bellamkonda

**Affiliations:** ^1^School of Electrical and Computer Engineering, Georgia Institute of Technology, Atlanta, GA, United States; ^2^Department of Biomedical Engineering, Pratt School of Engineering, Duke University, Durham, NC, United States; ^3^Wallace H. Coulter Department of Biomedical Engineering, Georgia Institute of Technology and Emory University School of Medicine, Atlanta, GA, United States

**Keywords:** intracortical microelectrodes, blood–brain barrier, neuro-inflammatory response, chronic recordings, signal-to-noise ratio, correlation analysis

## Abstract

**Objective:**

The goal for this research was to identify molecular mechanisms that explain animal-to-animal variability in chronic intracortical recordings.

**Approach:**

Microwire electrodes were implanted into Sprague Dawley rats at an acute (1 week) and a chronic (14 weeks) time point. Weekly recordings were conducted, and action potentials were evoked in the barrel cortex by deflecting the rat’s whiskers. At 1 and 14 weeks, tissue was collected, and mRNA was extracted. mRNA expression was compared between 1 and 14 weeks using a high throughput multiplexed qRT-PCR. Pearson correlation coefficients were calculated between mRNA expression and signal-to-noise ratios at 14 weeks.

**Main results:**

At 14 weeks, a positive correlation between signal-to-noise ratio (SNR) and NeuN and GFAP mRNA expression was observed, indicating a relationship between recording strength and neuronal population, as well as reactive astrocyte activity. The inflammatory state around the electrode interface was evaluated using M1-like and M2-like markers. Expression for both M1-like and M2-like mRNA markers remained steady from 1 to 14 weeks. Anti-inflammatory markers, CD206 and CD163, however, demonstrated a significant positive correlation with SNR quality at 14 weeks. VE-cadherin, a marker for adherens junctions, and PDGFR-β, a marker for pericytes, both partial representatives of blood–brain barrier health, had a positive correlation with SNR at 14 weeks. Endothelial adhesion markers revealed a significant increase in expression at 14 weeks, while CD45, a pan-leukocyte marker, significantly decreased at 14 weeks. No significant correlation was found for either the endothelial adhesion or pan-leukocyte markers.

**Significance:**

A positive correlation between anti-inflammatory and blood–brain barrier health mRNA markers with electrophysiological efficacy of implanted intracortical electrodes has been demonstrated. These data reveal potential mechanisms for further evaluation to determine potential target mechanisms to improve consistency of intracortical electrodes recordings and reduce animal-to-animal/implant-to-implant variability.

## Introduction

Brain machine interfaces (BMIs) using intracortical electrodes are promising to restore virtual and physical functionality to paralysis patients (Simeral et al., 2011; Collinger et al., 2013; Perge et al., 2014; Bouton et al., 2016; Downey et al., 2016; Ajiboye et al., 2017). However, reduction in amplitude and number of recorded spikes directly impacts the accuracy of machine control (Perge et al., 2014). Signal loss in both clinical (Simeral et al., 2011; Collinger et al., 2013; Perge et al., 2014; Bouton et al., 2016; Downey et al., 2016; Ajiboye et al., 2017) and preclinical (Karumbaiah et al., 2013; Saxena et al., 2013; Kozai et al., 2015; Nolta et al., 2015; Sharma et al., 2015; McCreery et al., 2016) intracortical electrode models have been well documented. A potential biological cause is chronic neurodegeneration, which has been characterized at the electrode–tissue interface (McConnell et al., 2009; Potter-Baker et al., 2015). Additionally, histology of neuronal nuclei density has been significantly correlated with signal-to-noise ratio (SNR) at the time of sacrifice (~300 days) (McCreery et al., 2016).

Previous work has suggested that the severity and duration of chronic blood–brain barrier (BBB) breach may influence chronic recordings (Potter et al., 2012; Saxena et al., 2013; Nolta et al., 2015; Kozai et al., 2016). The results have shown a negative correlation between IgG localization (a circulatory macromolecule) at the electrode interface and SNR (Karumbaiah et al., 2013; Saxena et al., 2013; Nolta et al., 2015). IgG accumulation has also shown to significantly and inversely correlate with impacts on behavioral motor function following electrode implantation in the motor cortex (Goss-Varley et al., 2017). While IgG localization demonstrates BBB leakage, it provides no information on how the BBB has been breached. Here, we investigate the molecular sequelae to implanted intracortical electrodes in the context of SNR to identify possible contributors to recording success.

For this study, key markers of BBB dysregulation, macrophage phenotype, and neuronal health at the mRNA level were quantified following electrode implantation. Animal-to-animal recording variability was leveraged to analyze correlations with mRNA expression at a chronic (14 week) time point to better elucidate potential mechanisms associated with electrode failure. To achieve this objective, functional microwire electrodes were implanted into the rat barrel cortex acutely (for 1 week) and chronically (for 14 weeks). At each endpoint, mRNA was extracted for Fluidigm multiplex qRT-PCR analysis. The calculated fold changes for each animal were compared to its functional recordings *via* a Pearson coefficient correlation. Primers for neuroinflammation, BBB integrity, innate inflammation, and leukocyte infiltration were investigated (see Table 1).

**Table 1:** Overview of significant Pearson correlation at 14 weeks for (A) neuroinflammation markers, (B) blood–brain barrier (BBB) markers, (C) leukocyte infiltration markers, and (D) inflammation markers.

Groups	Primers	Pearson correlation (*p* < 0.05)
Neuro-inflammtion	CD68	No
	GFAP	Yes
	NeuN	Yes

BBB	claudin-5	No
	occludin	No
	zona-occludens-1	No
	cdh5	Yes
	PDGFR-β	Yes
	AQP-4	No

Leukocyte adhesion	CD45	No
	ACAM	No
	ICAM1	No
	ICAM2	No
	sel-e	No
	sel-p	No
	VCAM1	No

Inflammation M1-like	CCR7	No
	CD32	No
	CD64	No
	CD80	No
	CD86	No

Inflammation M2-like	Arg-1	No
	CD163	Yes
	CD206	Yes

## Materials and Methods

### Surgical Preparation and Electrode Implantation

**Figure 1:**
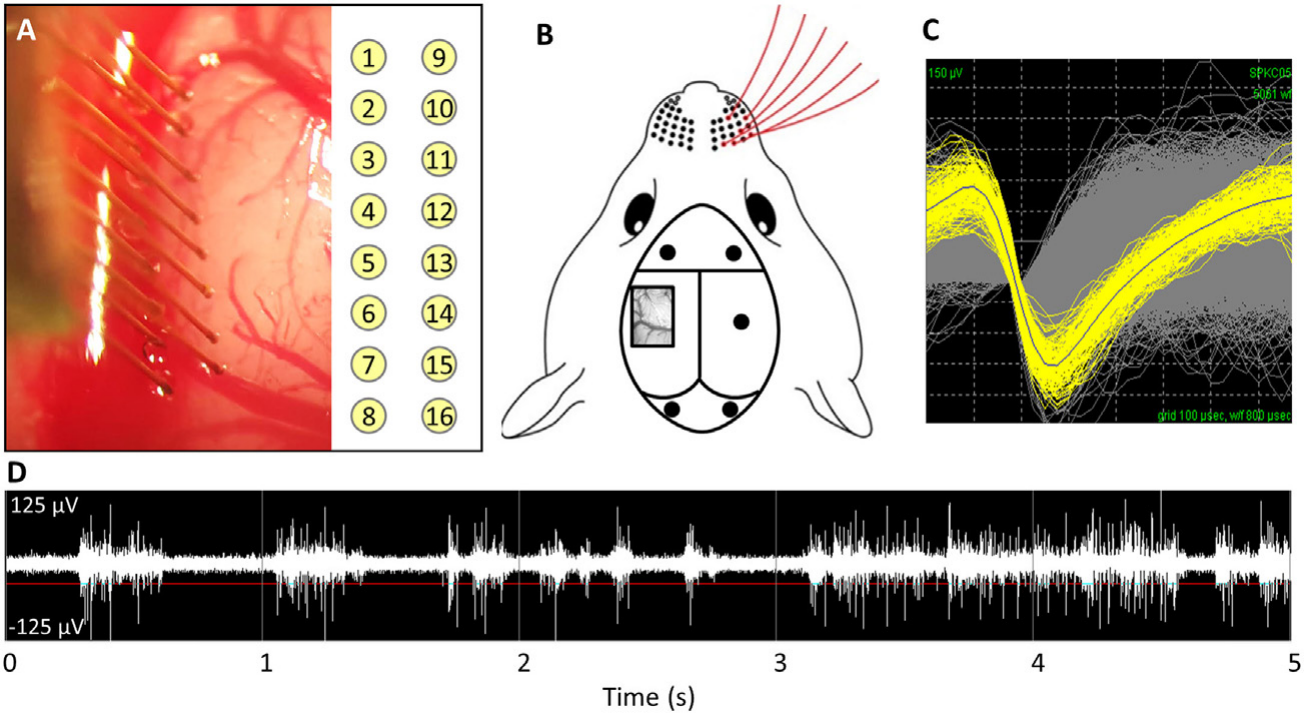
**(A)** Implantation of microwire array and electrode site map. **(B)** Representative image of barrel cortex craniotomy and anchoring/grounding screws. **(C)** Average waveforms for a single unit. **(D)** Acquired raw waveforms from recording system with example threshold setting.

This study was carried out in accordance with the recommendations of the Institutional Animal Care and Use Committee (IACUC) at the Georgia Institute of Technology. The protocol was approved by the Georgia Institute of Technology. Adult male Sprague Dawley rats (250–300 g) were implanted for 1 week (*n* = 5) or 14 weeks (*n* = 6). The implanted electrodes were polyimide coated tungsten microwires (Tucker-Davis Technologies, FL, USA). The array had 16 electrodes arranged in a 2 × 8 pattern spaced 300 µm apart in the *x*-direction and 500 µm apart in the *y*-direction (see Figure 1A). The electrodes were 50 µm in diameter and 5 mm in length. All microwires were sterilized by ethylene oxide and degassed for 12 h. Each rat was anesthetized with 2% isoflurane, and their head was shaved and sterilized with chlorohexidine and isopropanol. Each rat’s head was stereotaxtically positioned and a subcutaneous injection of lidocaine (Henry Schein, NY, USA) was administered locally prior to incision. Following a midline incision, the periosteum was scraped away and etch gel (Henry Schein, NY, USA) was applied to the skull. Holes for the anchoring screws were then drilled (2 anterior to bregma, 2 posterior to lambda, and 1 opposite the craniotomy), and five screws were inserted (see Figure 1B). A 3 mm × 5 mm craniotomy was drilled at 1.5 mm posterior from bregma and 4 mm lateral from the midline (see Figure 1B). The dura was retracted using a bent 25-gage needle and bleeding was controlled using gel foam (Pfizer, NY, USA) soaked with sterile saline. Grounding wires were wrapped around the anchoring screws prior to insertion. Each array was implanted at a 15° angle to a depth of 1,200 µm, targeting the IV cortical layer of the barrel cortex. Sterile 1.5% SeaKem agarose (Lonza, NJ, USA) was applied above the craniotomy and UV curing dental cement (Henry Schein, NY, USA) was used to secure the electrodes to the anchor screws and the skull. The incision was wound clipped and animals were injected intramuscularly with buprenorphine. Animals received daily subcutaneous injections of antibiotic, Baytril (Bayer, PA, USA), for 2 weeks.

### Electrophysiology and Analysis

**Figure 2:**
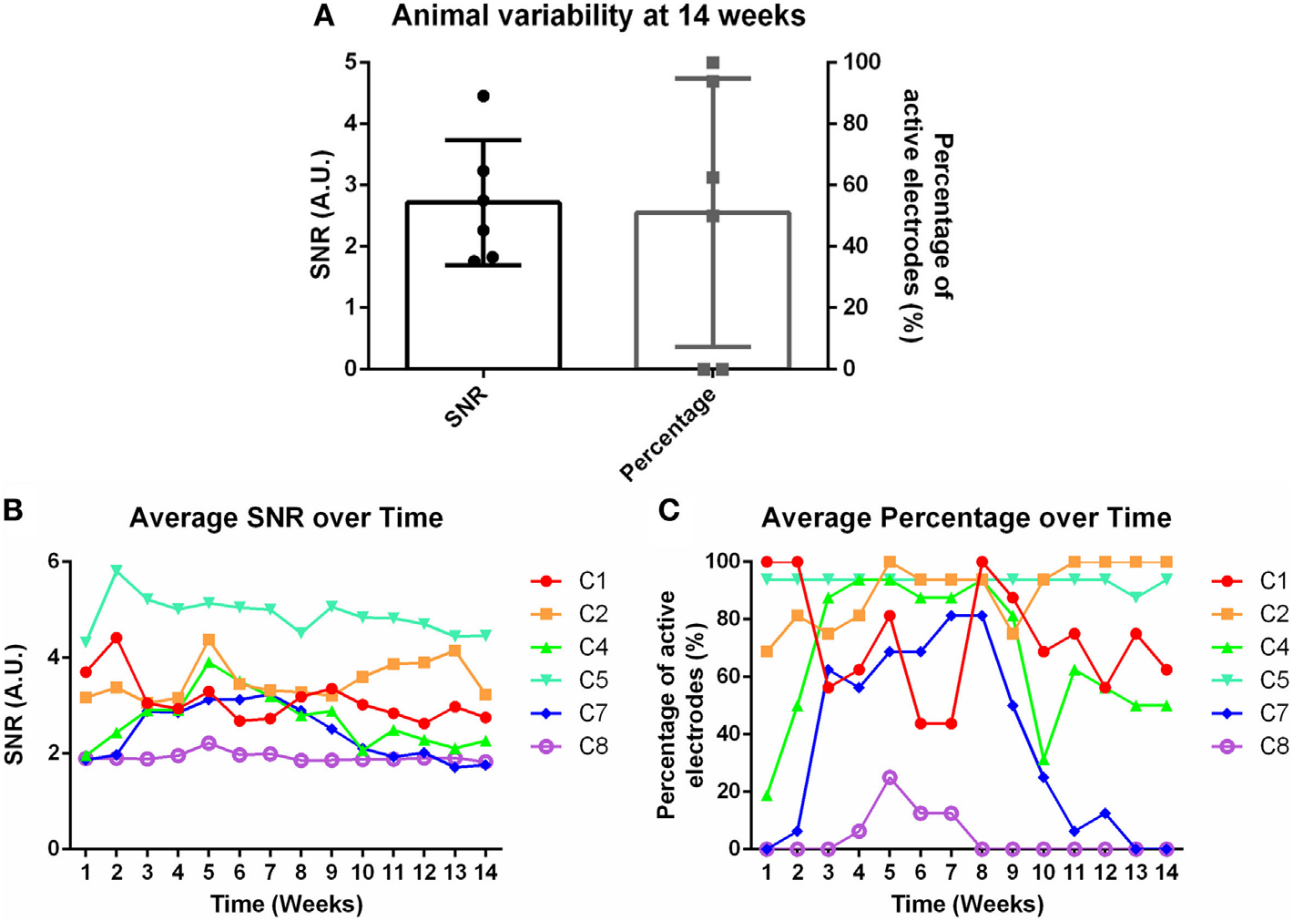
**(A)** Animal variability at 14 weeks demonstrated through signal-to-noise ratio (SNR) and percentage of active electrodes. **(B)** Average SNR and **(C)** average percentage of active electrodes over time for individual animals.

Weekly recordings were collected with a 32-channel data-acquisition system (Plexon, TX, USA). Signals were amplified at 1,000 × gain, band-pass filtered at 500–5,000 Hz and sampled at 40 kHz. Animals were anesthetized with ketamine/xylazine/acepromazine cocktail as isoflurane suppresses cortical firing in the barrel cortex. For each recording session, two files where recorded: (1) an evoked file in which the rat’s whiskers were deflected for ~1 min, generating action potentials (see Figures 1C,D) and (2) a noise file in which no signals were evoked for 10 s. In Offline Sorter (Plexon, TX, USA), the channels in the evoked files were thresholded at −4σ (standard orders of deviation), and units were sorted using K means cluster cutting. To verify units, the interspike interval histogram was analyzed and the presence of a clear refractory period was observed for a unit to be declared. Units that had fewer than 100 action potentials were excluded. Spontaneous action potentials were removed from the noise file. Sorted files were then exported into Matlab and custom code was used to calculate the SNR by dividing the peak-to-peak voltage (V_p-p_) by two times the SD of noise (Eq. 1) (Nordhausen et al., 1996; Srinivasan et al., 2016).

(1) $$\text{SNR}=\frac{v_{p-p}}{2{\text{*Std}}_{\text{noise}}}=\frac{\left(V_{\max}-V_{\min}\right)}{2\text{*}\sqrt{\frac{\sum {\left({\text{noise}}_{\text{j}}-\mu_{\text{noise}}\right)}^{2}}{\left(n-1\right)}}}$$

### qRT-PCR and Analysis

At the designated time point, animals were transcardially perfused with sterile PBS (200 mL). The electrodes and headcap were removed and the brain was extracted. A 4-mm biopsy punch was taken at a depth of 2 mm around the electrode implant site. The biopsy punch was immediately frozen in liquid nitrogen and stored at −80°C. Age-matched naïve animals were sacrificed in the same manner as well and the 4-mm biopsy punch was removed from a depth of 2 mm at the same location in the brain. Total RNA was extracted using the RNeasy Plus Universal Kit (Qiagen, CA, USA). RNA integrity was assessed with the Agilent Bioanalyzer using Agilent RNA 6000 Nano Kit (Agilent Technologies, CA, USA), and purity was assessed with the Nanodrop 8000 Spectrophotometer (Thermo Fisher Scientific, MA, USA). For all samples, RNA integrity numbers were above 7, 260/280 were above 1.8, and 260/230 were above 1.0. cDNA was synthesized using the Fluidigm Reverse Transcription kit (#100-6298) (Fluidigm, CA, USA). A 96 qRT-PCR assay using the Fluidigm Biomark HD (Fluidigm, CA, USA) was run in triplicate for each sample using the Duke Center for Genomic and Computational Biology. The Delta Gene Assays (Fluidigm, CA, USA) were designed using the D3 Assay Design (Fluidigm, CA, USA). CT values were averaged together across triplicates. ΔCT values were calculated by subtracting the geometric mean of four housekeeping genes (GAPDH, HRPT, SDHA, RPL13A) from each CT value. ΔΔCT values were calculated by subtracting the arithmetic average of the naïve samples from the ΔCT values. All statistics were performed in the ΔΔCT space. Fold change was then calculated by taking the base 2 exponent of—ΔΔCT. A Bonferroni sequential correction (Benjamini and Hochberg, 1995) was applied to a Student’s *t*-test to determine significance between 1- and 14-week microwire animals.

### Correlation Analysis

To correlate the relation between average SNR and mRNA fold change, a Pearson correlation coefficient (*r*) with a *p*-value was calculated in Matlab. The electrode SNRs for each animal were averaged at each timepoint. The 14-week SNRs were compared with the mRNA extracted at 14 weeks for each animal.

### Immunohistochemistry

At 1 week, rats implanted with microwires were transcardially perfused with PBS, 4% paraformaldehyde, and 20% sucrose. Following decapitation, the skulls were exposed and placed in 4% paraformaldehyde overnight at 4°C and then 30% sucrose overnight at 4°C. The brains were then extracted and stored in 30% sucrose at 4°C overnight or until the brains sunk to the bottom of the container. Brains were frozen at −20°C and cryosectioned transversely onto charged glass slides (VWR, PA, USA). Slides were thawed to room temperature and washed with PBS. The slides were incubated at room temperature in blocking solution (0.4% Triton-X, 4% goat serum in PBS) for 1 h. The following primary antibodies were used: rabbit anti-GFAP (1:1,000, DAKO, CA, USA), mouse IgG1 anti-NeuN (1:500, Millipore, CA, USA), and mouse anti-CD68 (1:500, Millipore, CA, USA). Primary antibodies were diluted in blocking solution and incubated overnight at 4°C. Slides were then washed in PBS and washing solution (0.4% Triton-X in PBS). The appropriate secondary antibody was applied for 1 h at room temperature, followed by DAPI for 15 min. Slides were washed again in PBS and washing solution, dried, and coverslipped with Fluoromount-G (Southern Biotech, AL, USA). Stained slides were imaged at 10× on a Zeiss Axiovert 200 M (Carl Zeiss, NY, USA).

## Results

### Animal-to-Animal Variability in Electrophysiology

Weekly recordings were conducted in the barrel cortex. Rats were anesthetized and whiskers were deflected to generate evoked potentials. SNRs were calculated for each electrode within each time point within each animal. Eight rats were implanted for the chronic time point, but two were removed from the study due to headcap failure (C3 and C6). Figure 2A demonstrates the animal-to-animal variability present at 14 weeks for both SNR and percentage of active electrodes. Figures 2B,C shows the average SNR and percentage of active electrodes plots, subsequently, for each individual animal over time. A three-way nested ANOVA was run in Matlab on the SNR and electrode percentage data. Over time, no significant change was observed for either metric (*p* > 0.05). However, animal variability in SNR and percentage of active electrodes was significant. This variability was used to investigate possible correlations with underlying molecular differences through mRNA expression. Briefly, Pearson correlation coefficients and *p*-values were calculated for each mRNA primer and the corresponding animal’s SNR. Pearson correlation coefficients were considered significant when the *p*-value ≤ 0.05. Additionally, mRNA expression was compared between 1 and 14 weeks using a Bonferroni sequential corrected Student’s *t*-test in which significance was determined when the *p*-value was ≤0.05.

### Neuroinflammation

**Figure 3:**
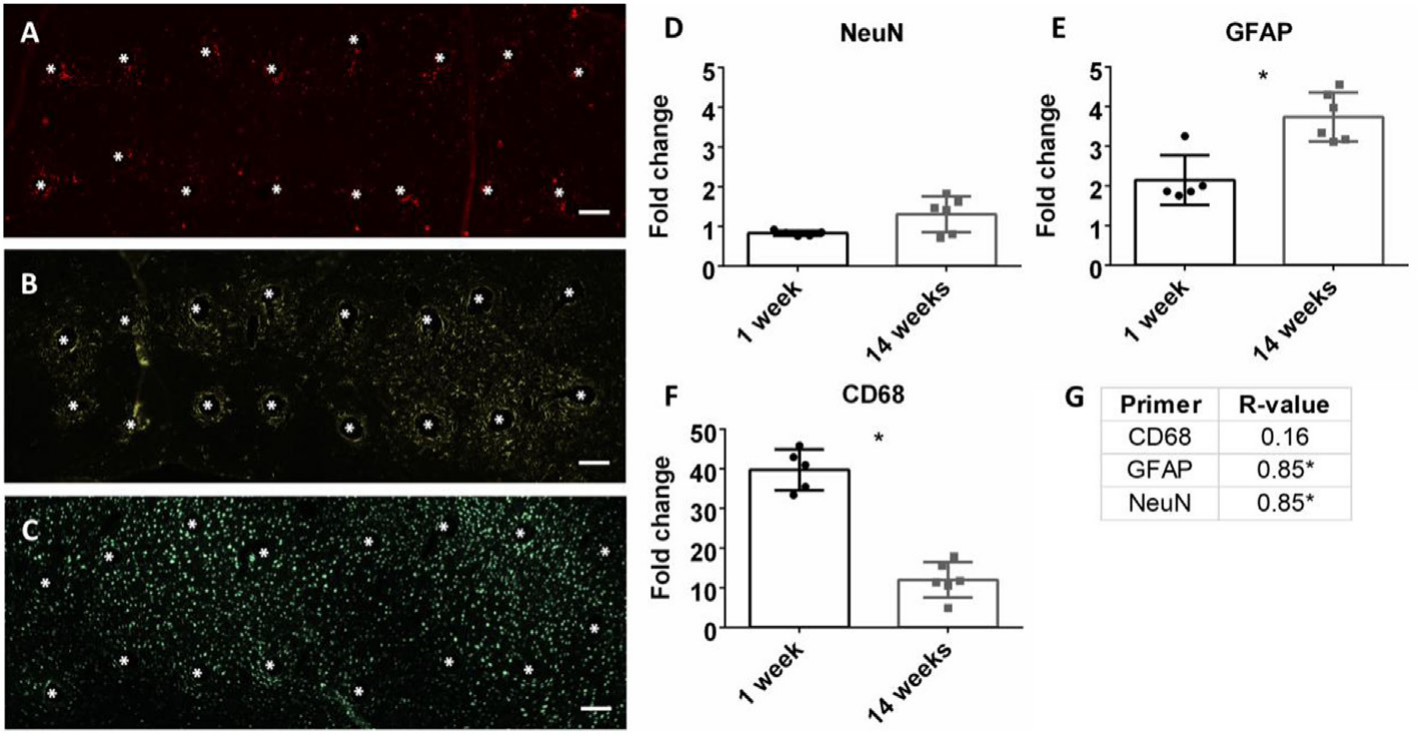
Representative images of 16 electrode microwire arrays at 1 week with * representing electrode location for **(A)** CD68, **(B)** GFAP, and **(C)** NeuN antibody staining (scale bar = 100 µm). Fold change comparison between 1 and 14 weeks for **(D)** NeuN, **(E)** GFAP, and **(F)** CD68 (*p < 0.05, Student’s *t*-test, Bonferroni corrected). Each time point was compared to age-matched naïve controls to calculate fold change. **(G)** Pearson correlation values for CD68, GFAP, and CD68 (*p < 0.05).

At the conclusion of each time point, mRNA was extracted from biopsied brain tissue and mRNA expression was calculated. Neuroinflammation markers, classically found in the intracortical electrode implant literature, were analyzed. This included CD68 for activated microglia/macrophages, GFAP for astrocytes, and NeuN for neuronal nuclei (Polikov et al., 2005; Potter et al., 2012; Saxena et al., 2013; Sawyer et al., 2014; Nolta et al., 2015). Representative immunohistochemistry images of CD68, GFAP, and NeuN at the microwire interface are shown in Figures 3A–C at 1 week. There was a significant reduction of CD68 expression from 1 to 14 weeks, and a significant increase of GFAP from 1 to 14 weeks (*p* < 0.05) (Figures 3E,F). No change was observed for NeuN expression (Figure 3D). At 14 weeks, the Pearson correlation coefficient was positively significant for both NeuN and GFAP (*r* = 0.85, *p* < 0.05, and *r* = 0.85, *p* < 0.05, Figure 3G; Figures S1A,B in Supplementary Material). As NeuN marks neuronal nuclei, the positive correlation with SNR is suggestive that SNR represents neuronal population at the electrode interface.

### Inflammation Milieu

**Figure 4:**
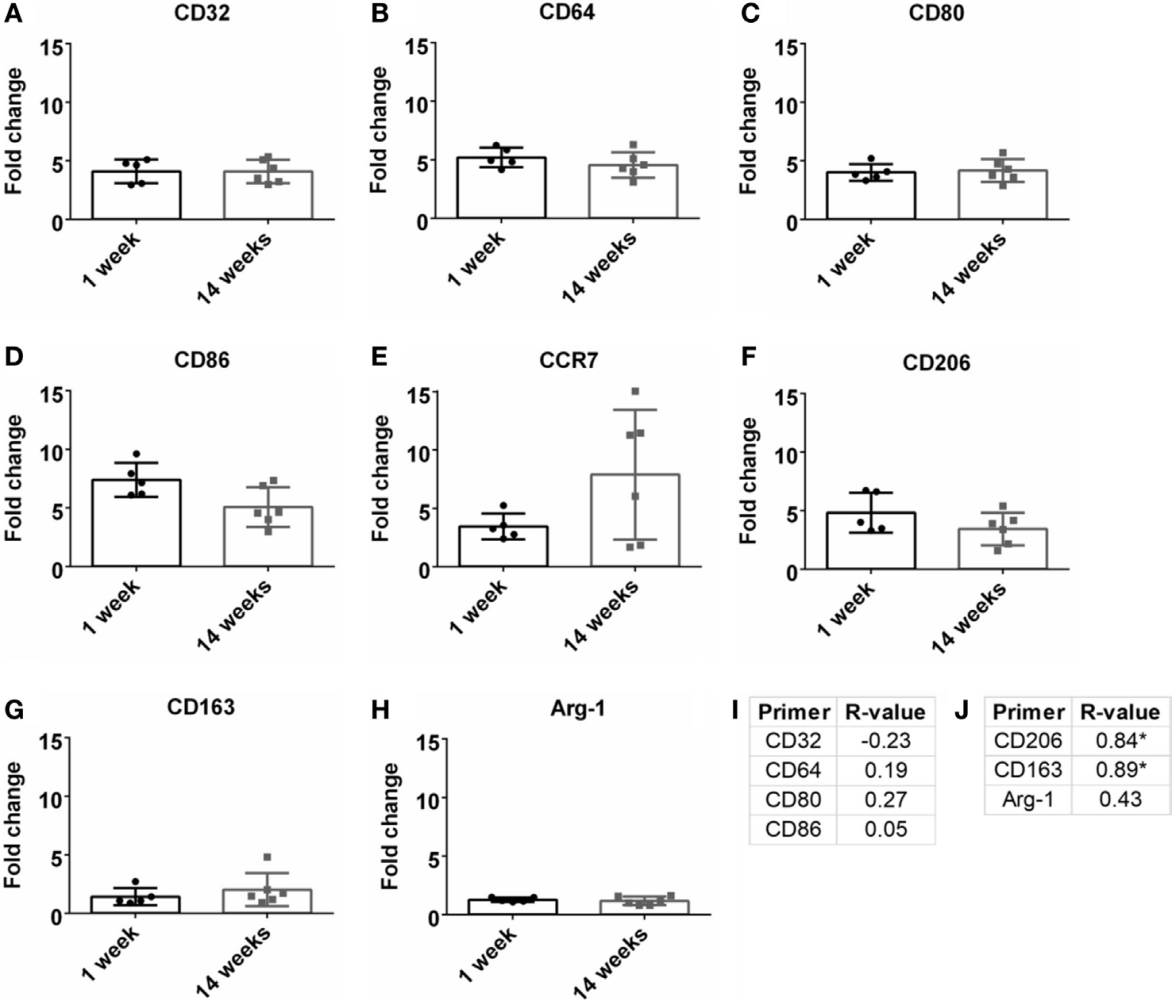
Fold change comparisons between 1 and 14 weeks for M1-like pro-inflammatory markers **(A)** CD32, **(B)** CD64, **(C)** CD80, **(D)** CD86, and **(E)** CCR7, and M2-like anti-inflammatory markers **(F)** CD206, **(G)** CD163, and **(H)** Arg-1 (*p < 0.05, Student’s *t*-test, Bonferroni corrected). Each time point was compared to age-matched naïve controls to calculate fold change. Pearson correlations for **(I)** M1-like and **(J)** M2-like markers (*p < 0.05).

Macrophages are a strong part of the wound healing and neuroinflammatory response (Strauss-Ayali et al., 2007; Kigerl et al., 2009; David and Kroner, 2011; Mokarram et al., 2017). Markers for M1-like or pro-inflammatory response were analyzed and were upregulated at both 1 and 14 weeks (Figures 4A–E). However, there was no significant change over time. No significance was found for Pearson correlation coefficients for pro-inflammatory markers at 14 weeks (Figure 4I; Figure S1C in Supplementary Material). For M2-like markers or anti-inflammatory response (Figures 4F–H), CD206 and CD163 had a significant positive Pearson correlation coefficient at 14 weeks (*r* = 0.84, *r* = 0.89, *p* < 0.05, Figure 4J; Figure S1D in Supplementary Material).

### Vascular Integrity/BBB Breach Status

**Figure 5:**
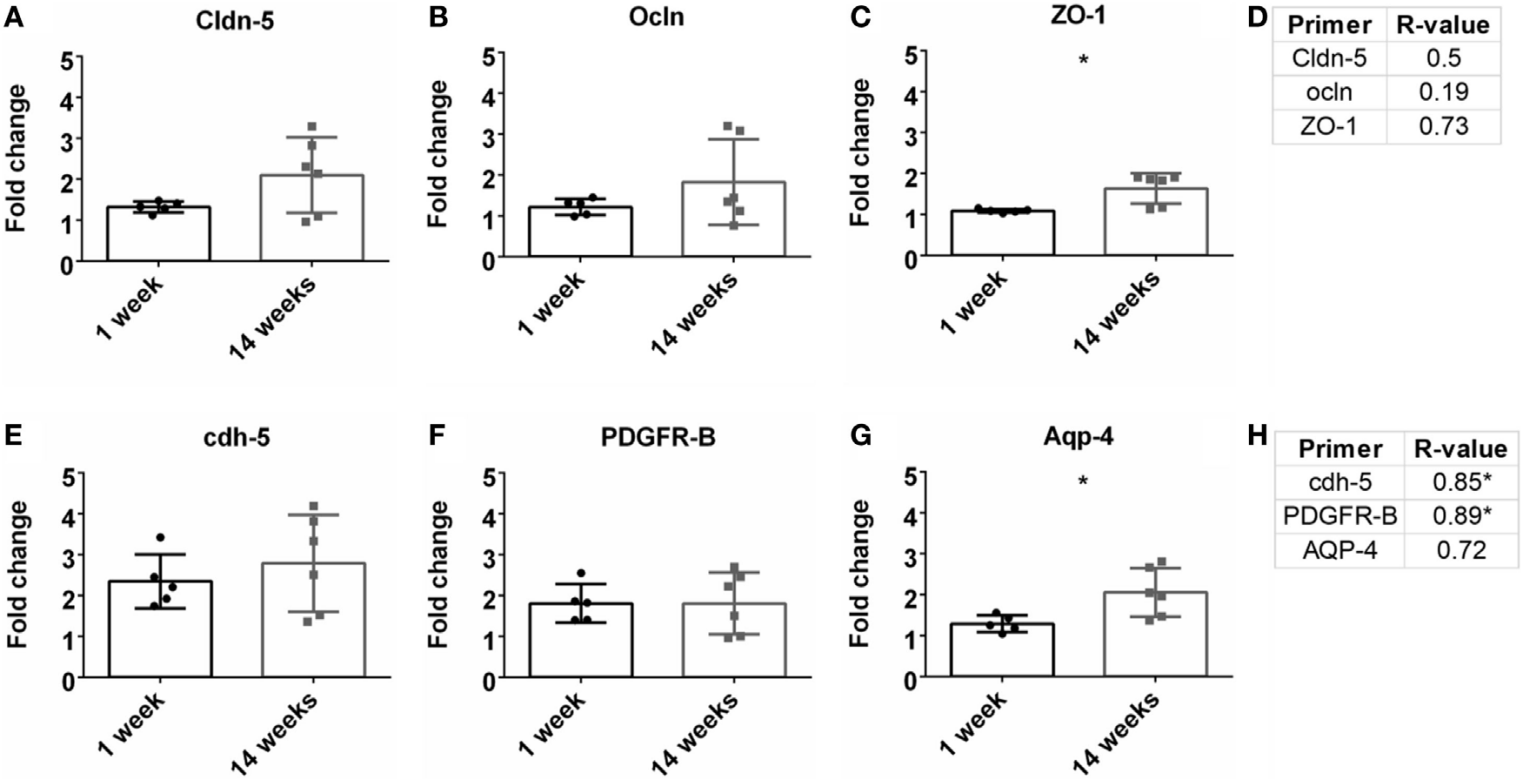
Fold change comparisons between 1 and 14 weeks for tight junction proteins **(A)** Cldn-5, **(B)** occluding (Ocln), and **(C)** zona-occludens-1 (ZO-1) and other blood–brain barrier (BBB) markers, **(E)** cdh-5, **(F)** PDGFR-β, and **(G)** AQP-4 (*p < 0.05, Student’s *t*-test, Bonferroni corrected). Each time point was compared to age-matched naïve controls to calculate fold change. Pearson correlations for **(D)** tight junction protein markers and **(H)** other BBB markers (*p < 0.05).

Previous research has demonstrated the importance of BBB and vasculature to neuronal health (Abbott et al., 2006; Ivens et al., 2007; Stolp and Dziegielewska, 2009; Zlokovic, 2011; Obermeier et al., 2013; Ryu et al., 2015). Here, tight junction proteins and additional BBB markers were observed. First, common tight junction proteins, zona-occludens-1 (ZO-1), claudin-5 (cldn5), and occludin (ocln) were evaluated to assess BBB fidelity. ZO-1 had a significant upregulation at 14 weeks compared to 1 week (Figure 5C). No other significant changes from 1 to 14 weeks were observed for cldn5 or ocln (Figures 5A,B). No significant Pearson coefficient correlation was found for cldn5, ocln, or ZO-1 at 14 weeks (Figure 5D; Figure S1E in Supplementary Material). With no significant correlations with tight junction proteins, additional BBB markers were next evaluated. These included cell-to-cell junctions, VE-cadherin (cdh-5), pericytes (PDGFR-β), and astrocyte end-feet (Aqp-4). Aqp-4 was significantly upregulated at 14 weeks compared to 1 week (Figure 5G). Expression levels remained the same for cdh-5 and PDGFR-β (Figures 5E,F). There was a significant positive Pearson correlation coefficient at 14 weeks for cdh-5 and PDGFR-β (*r* = 0.85, *r* = 0.89, *p* < 0.05, Figure 5H; Figure S1F in Supplementary Material). However, no significant correlation was observed for Aqp-4.

### Leukocyte Recruitment and Adhesion

**Figure 6:**
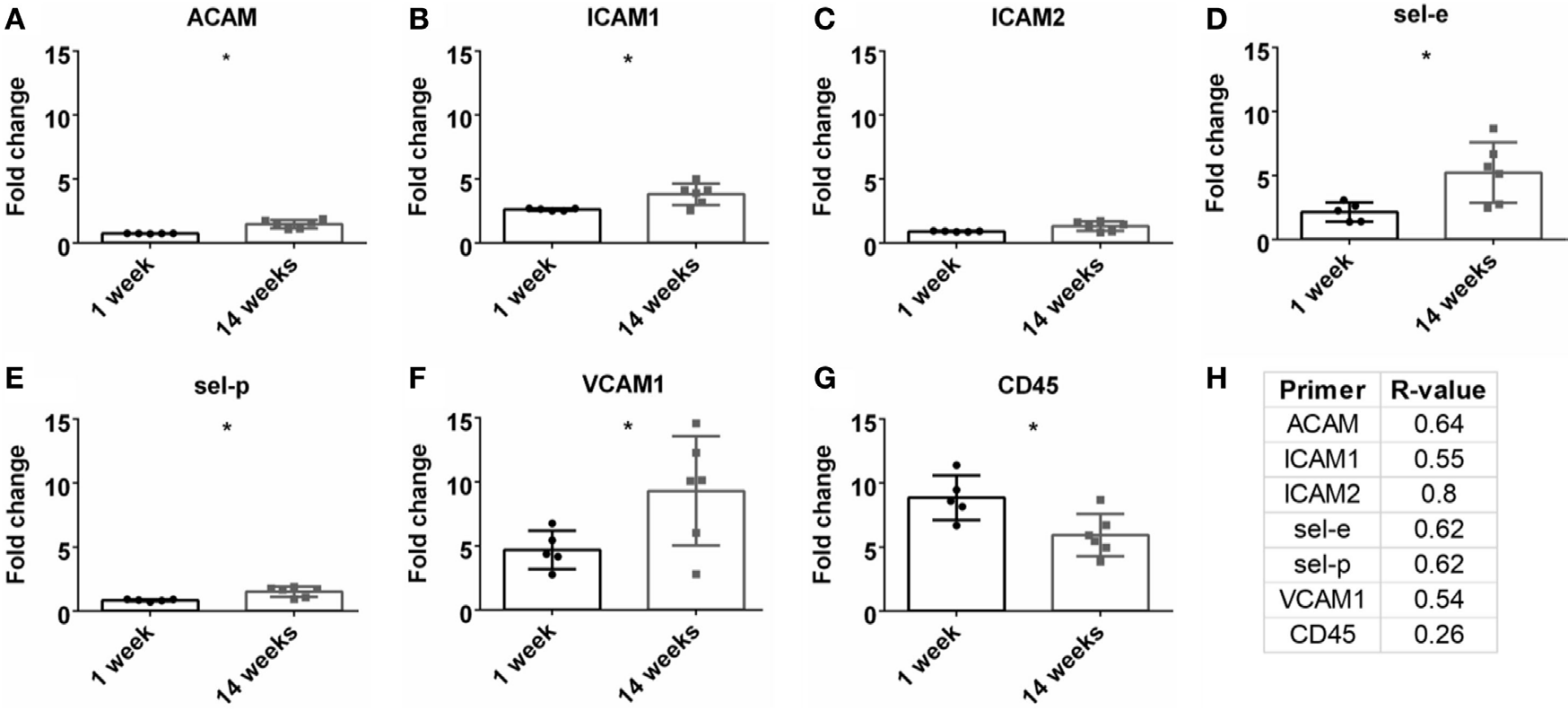
Fold change comparisons between 1 and 14 weeks for endothelial adhesion markers **(A)** ACAM, **(B)** ICAM1, **(C)** ICAM2, **(D)** sel-e, **(E)** sel-p, **(F)** VCAM1 and pan-leukocyte marker, **(G)** CD45 (*p < 0.05, Student’s *t*-test, Bonferroni corrected). Each time point was compared to age-matched naïve controls to calculate fold change. **(H)** Pearson correlation for endothelial adhesion and pan-leukocyte markers (*p < 0.05).

A detrimental outcome of BBB leakage is the infiltration of leukocytes (Greenwood et al., 2011; Obermeier et al., 2013). This can be monitored by leukocyte cell markers and endothelial cell adhesion markers. The fold change for the pan-leukocyte marker (CD45) was analyzed and expression significantly decreased at 14 weeks compared to 1 week (*p* < 0.05, Figure 6G). However, no significant Pearson correlation was observed with SNR (Figure 6H). A variety of adhesion markers were analyzed (ACAM, ICAM1, ICAM2, sel-e, sel-p, VCAM1, Figures 6A–F). All were significantly upregulated at 14 weeks (except for ICAM2), suggesting increased leukocyte extravasation, but again, no significant Pearson correlation was found (Figure 6H; Figure S1G in Supplementary Material).

## Discussion

If BMIs are to be successful, the signal from the intracortical electrode (i.e., the input) must be able to reliably and robustly record for long durations (on the order of years). Previously, a relation between BBB integrity and electrode performance has been demonstrated (Saxena et al., 2013), and here, these findings have been extended *via* an investigation of the underlying molecular mechanisms. Penetration and destruction of vessels during implantation may explain electrode recording variability per animal, and the importance of vascular integrity has been implicated in several studies in regards to neural health and electrodes (Kozai et al., 2010; Shih et al., 2012; Saxena et al., 2013; Nolta et al., 2015). However, the direct mechanisms driving BBB dysregulation and electrode failure are not well understood. These data have shown a positive correlation between SNR and different molecular targets, and this information could be further investigated to evaluate the importance in relation to electrode failure.

Expression levels for common neuroinflammation markers were evaluated. As seen in Table 1 and Figures 3D,E, significant positive correlation was observed for NeuN and GFAP, but not for CD68. Previous electrode literature has suggested that the development of the astroglial scar at the electrode tissue interface is the primary cause for signal degeneration (Polikov et al., 2005). This attitude was pervasive through other neurodegenerative fields; however, this view has begun to change. The Sofroniew lab has demonstrated the importance of astrocyte support in a spinal cord injury model and through knock-out models, when the astrocytic scar is ablated, axonal regeneration is in fact impaired (Anderson et al., 2016). McCreery et al. (2016) conducted an analysis with Utah electrodes implanted in the cat sensorimotor cortex for almost a year. Histology was correlated with electrophysiology using the Pearson correlation, and a significant positive correlation was found for both NeuN and GFAP within 80 µm of the electrode for signal amplitudes at the experiment endpoint. Our data corroborate McCreery’s findings, suggesting that presence of GFAP+ astrocytes is positively correlated with increased SNR. Therefore, developing treatment strategies to improve astrocyte recruitment (as opposed to inhibiting astrocytes) may prove beneficial for chronic intracortical implants.

The influence of M1-like and M2-like environments on neural health has been an area of study within the central and peripheral nervous systems (Kigerl et al., 2009; David and Kroner, 2011; Mikita et al., 2011; Mokarram et al., 2012; Vogel et al., 2013; Cherry et al., 2014; Sawyer et al., 2014; Kim et al., 2016; Tang and Le, 2016). With BBB breach following disease or injury, the influx of innate monocytes and macrophages can influence the neurological outcomes (Kigerl et al., 2009; Mikita et al., 2011). Common M1-like (CCR7, CD32, CD64, CD80, CD86) and M2-like (Arg-1, CD163, CD206) markers were evaluated to determine the relation between inflammation and SNR (Table 1; Figure 4). At 14 weeks, M2-like CD163 and CD206 were significantly positively correlated with SNR. CD163 is a general receptor found on all subsets M2-like macrophages, while CD206 is specific for M2a and M2c, which is responsible for tissue repair and pro-healing functions (David and Kroner, 2011; Mokarram et al., 2012). Research from the spinal cord (Kigerl et al., 2009) and the peripheral nerve (Mokarram et al., 2017) have demonstrated the benefits of a M2-like macrophage environment for neural health and repair. Additionally, non-functional electrodes implanted in a bone marrow chimera mouse model showed blood-borne macrophage accumulation at 16 weeks (Ravikumar et al., 2014). It would be beneficial to investigate if the M2 macrophage theory also holds true for functional recordings from the cortex.

To investigate the status of the BBB, tight junction protein expression was analyzed (Table 1; Figures 5A,B). Tight junctions are crucial to maintaining a healthy, intact BBB, and loss can lead to neurodegeneration (Kanda et al., 2004; Abbott et al., 2006; Zhong et al., 2008; Argaw et al., 2009; Henkel et al., 2009; Liu et al., 2012; Paul et al., 2013). Interestingly, no significant correlations were observed for these tight junction expressions. Additional components of the BBB were then investigated, including AQP-4, cdh5, and PDGFR-β (Table 1; Figures 5C,D). While there was a significant correlation with GFAP expression, there was no correlation with AQP-4, which is a common marker on astrocyte end-feet interacting with the BBB. VE-cadherin (cdh5) had a significant positive correlation with the SNR. VE-cadherin is a cell-to-cell junction for endothelial cells, and the removal of VE-cadherin severely weakens the BBB (Wallez and Huber, 2008; Giannotta et al., 2013; Tietz and Engelhardt, 2015). Thus far, no work has been done confirming the impact of VE-cadherin loss on neurodegeneration. Further work exploring the potential connection between VE-cadherin loss and its impact on neurodegeneration could be of interest.

The data also show a significant positive correlation between PDGFR-β, a common pericyte receptor, and SNR (see Table 1; Figures 5C,D). Evaluation of PDGFR-β knockout mice demonstrated that vascular integrity in the brain was significantly compromised and became more susceptible to macromolecule leakage (Armulik et al., 2010). The Zlokovic lab built upon this work with the PDGFR-β knockout model showing that pericyte loss reduced cerebral blood flow and degraded BBB tight junction proteins. This resulted in neurodegeneration, and pericyte loss exacerbated amyloid-β clearance in Alzheimer’s disease models (Bell et al., 2010; Sagare et al., 2013; Halliday et al., 2016). The results from this study thus corroborate previously published data describing the importance of pericytes in the neurovascular unit and might suggest the importance of maintaining pericyte health to improve performance for intracortical electrodes.

A common cause/impact of BBB leakage is the increased expression of adhesion markers and leukocytes (Greenwood et al., 2011; de Vries et al., 2012; Obermeier et al., 2013; Shechter et al., 2013). Therefore, these markers were investigated in correlation with SNR (Table 1; Figures 6C,D). No significant correlation was found for leukocytes (CD45) or adhesion markers (ACAM, ICAM1, ICAM2, sel-e, sel-p, VCAM1). Elahy et al. (2015) demonstrated that loss of BBB integrity and inflammation does occur in an aging model, but no leukocytes were recruited. Others have shown that while leukocytes are recruited in different BBB leakage models, this cellular presence does not lead to neurodegeneration (Boztug et al., 2002; Shaftel et al., 2007). Our data may suggest that leukocyte infiltration is not a primary cause for neurodegeneration in an electrode implant model.

Overall, these data showed significant positive correlation between SNR and GFAP, VE-cadherin, and PDGFR-β. No significant correlations for leukocyte extravasation, inflammatory phenotypes, or tight junction expression were observed. This would suggest the importance of astrocytes (GFAP), adherens junctions (VE-cadherin), and pericytes (PDGFR- β) for maintaining strong SNR at chronic time points. These data offer insight into potential molecular mechanisms to explore for improving chronic intracortical recordings.

## Conclusion

The objective of this work was to better understand the molecular mechanisms influencing recording fidelity in electrode implant models. Previous work has suggested that BBB breach can influence chronic recordings. mRNA expression was correlated with SNR at a chronic (14 week) time point. Astrocytes, pericytes, and adherens junctions were identified as potential therapeutic targets to improve chronic intracortical recordings. Additional work with knock-out models and histological analysis is necessary to further validate the effect of these pathways. It is also important to remember that microwires were used for this study, and comparison to commonly used Michigan (research) and Utah (clinical) electrodes would be beneficial. This work provides direction for future studies and identification of BBB integrity markers that may influence and benefit chronic recordings in intracortical electrodes.

## Ethics Statement

This study was carried out in accordance with the recommendations of the Institutional Animal Care and Use Committee (IACUC) at the Georgia Institute of Technology. The protocol was approved by the Georgia Institute of Technology.

## Author Contributions

JF designed experiments, performed animal work, electrophysiology, data analysis, results interpretation, and wrote the manuscript. SC performed qRT-PCR and assisted with manuscript writing. TS assisted with experiment design, results interpretation, and manuscript writing. DM, AC, and VY assisted with animal work and data analysis. RB is the principal investigator.

## Conflict of Interest Statement

The authors declare that the research was conducted in the absence of any commercial or financial relationships that could be construed as a potential conflict of interest.
