# Supplementary figures and images for: Correlation of mRNA Expression and Signal Variability in Chronic Intracortical Electrodes
Source: Front Bioeng Biotechnol. 2018 Mar 27;6:26. doi: 10.3389/fbioe.2018.00026 (PMC5880884; doi:10.3389/fbioe.2018.00026)

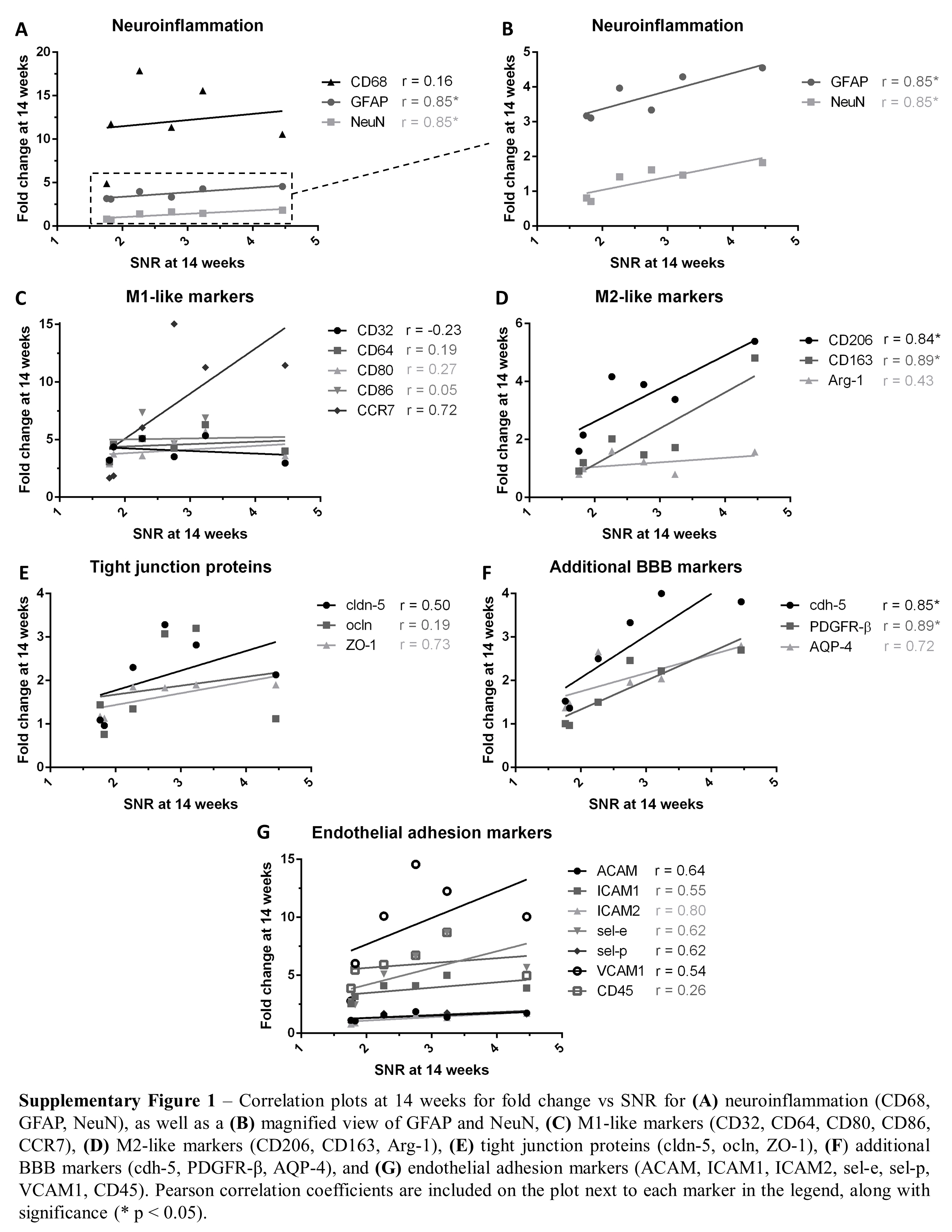

Supplement: Supplementary file 1 [file image_1.tif]
